# Isolated insular stroke: topography is the answer with respect to outcome and cardiac involvement

**DOI:** 10.3389/fneur.2024.1332382

**Published:** 2024-02-29

**Authors:** Fedra Kuris, Sara Tartaglia, Roberto Sperotto, Laura Ceccarelli, Daniele Bagatto, Simone Lorenzut, Giovanni Merlino, Francesco Janes, Carolina Gentile, Roberto Marinig, Lorenzo Verriello, Mariarosaria Valente, Giada Pauletto

**Affiliations:** ^1^Clinical Neurology Unit, Head-Neck and NeuroScience Department Udine, Udine University Hospital, Udine, Italy; ^2^Division of Neuroradiology, Diagnostic Imaging Department, Udine University Hospital, Udine, Italy; ^3^Neurology Unit, Head-Neck and NeuroScience Department, Udine University Hospital, Udine, Italy; ^4^Department of Medicine (DMED), University of Udine, Udine, Italy

**Keywords:** insular stroke, acute ischemic stroke, case series, good outcome, cardioembolism

## Abstract

**Background and purpose:**

Isolated insular strokes (IIS) are a rare occurrence due to the frequent concomitant involvement of adjacent territories, supplied by the M2 segment of the middle cerebral artery (MCA), and clinical aspects are sometimes contradictory. We aimed to describe clinical and radiological characteristics of a pure IIS case series, focusing on its functional outcome and cardiac involvement.

**Methods:**

We identified 15 isolated insular ischemic strokes from a pool of 563 ischemic strokes occurred between January 2020 and December 2021. Data collection consisted of demographic and baseline clinical characteristics, comorbidities, electrocardiograms, echocardiograms, stroke topography and etiology, reperfusive treatments, and outcome measures. Descriptive statistical analysis was carried out.

**Results:**

Newly detected cardiovascular alterations were the prevalent atypical presentation. Cardioembolism was the most frequent etiology. Most of patients had major neurological improvement at discharge and good outcome at 3-months follow-up.

**Discussion and conclusion:**

IIS are extremely rare, representing according to our study about 2.6% ischemic strokes cases per year, and patients have peculiar clinical manifestations, such as dysautonomia and awareness deficits. Our data suggest the possibility for these patients to completely recover after acute ischemic stroke notwithstanding the pivotal role of the insula in cerebral connections and the frequent association with MCA occlusion. Moreover, given the central role of the insula in regulating autonomic functions, newly detected cardiac arrhythmias must be taken into consideration, as well as a full diagnostic work-up for the research of cardioembolic sources. To our knowledge, this is the largest monocentric case series of IIS and it might be useful for future systematic reviews.

## 1 Introduction

The insular cortex, deeply located in the Sylvian fissure and separating temporal lobe from parietal and frontal lobes, is a highly connected integrative region, functionally divided into three zones: the posterior insular cortex (PIC), the middle insular cortex (MIC) and the anterior insular cortex (AIC). Afferences from the solitary nuclei and the spinal lamina I neurons project to the PIC, providing an interoceptive representation of the body physiological condition. This information is processed in the MIC and integrated with other inputs coming from multiple sources, in particular sensory cortex, that conveys emotional information from the external world, the cingulate cortex and the amygdala, providing homeostatic information on the motivational state. Finally, the inputs converge upon the AIC where new integrative processes are taking place; particularly auditory and language tasks activate the dorsal part of the AIC, motor tasks activate both anterior and posterior insula, and so on (1, 2).

This high level of integration may explain the extreme variety of manifestations in case of insular stroke. However, isolated insular strokes are rare, due to the frequent concomitant involvement of adjacent territories, supplied by the M2 segment of the middle cerebral artery (3).

For these reasons, clinical aspects of isolated insula strokes are sometimes contradictory, even if literature is homogeneous in describing a generally good outcome for isolated insular stroke (IIS) (4, 5).

In the present work, we aimed to describe clinical and radiological characteristics of pure ischemic stroke case series, trying to aggregate and correlate them according to the functional topography of the insula, and especially focusing on its functional outcome and cardiac involvement.

## 2 Methods

We retrospectively reviewed 563 patients diagnosed with first ever ischemic stroke, at the Stroke Unit of “Santa Maria della Misericordia” University Hospital of Udine (Italy), from January 1st, 2020, to December 31st, 2021.

We considered 113 patients with ischemic insular cortex involvement and, among them, we selected only isolated insular ischemic strokes (IIS), visually assessed by a board-certified neuroradiologist on non-contrast computerized tomography (NCCT) scans, acquired 24–48 h after the acute event or, when available, on diffusion-weighted magnetic resonance imaging (DWI or DW-MRI) scans.

Isolated insular lesions were clustered based on the involvement of anterior insular cortex (AIC), posterior insular cortex (PIC), and total insular cortex (TIC).

Data collection from medical charts included demographic and baseline clinical characteristics, comorbidities, including the presence of leukoaraiosis-defined as the presence of hypodense periventricular white-matter lesions at CT scan (4), stroke severity measured by the incoming National Institute of Health Stroke Scale (NIHSS) and initial disability measured by the incoming modified Rankin Scale (mRS), electrocardiogram (ECG), and echocardiogram characteristics. Cardiac rhythm monitoring with 12-lead ECG lasted meanly 48 h.

We defined patients’ clinical presentation according to the latest comprehensive literature review on strokes confined to the insula by Di Stefano et al. (5) We categorized motor and somatosensory deficits, dysarthria, aphasia, and vestibular-like syndrome as “typical” symptoms, whereas dysphagia, awareness deficits, gustatory disturbances, dysautonomia, neuropsychiatric or auditory disturbances and headache were categorized as “atypical” presentations.

Stroke etiology was defined according to the TOAST classification (6). In-hospital therapies and procedures data included antihypertensive and antiarrhythmic infusive therapies, intravenous thrombolysis (IVT) with tissue-type plasminogen activator (rT-PA) and mechanical thrombectomy (MT). Outcome measures included NIHSS and mRS at discharge, in-hospital mortality, major neurological improvement at discharge (improvement of 8 points on the NIHSS from baseline or a NIHSS score of 0 or 1 at discharge), 3-months-good-outcome measured by mRS of 0–2 and 3-months-mortality. Good outcome was defined as mRS of 0–2 to include patients with functional independence 3 months after stroke, in line with previous stroke outcome studies, according to the definition of mRS of 2 (“slight disability; unable to carry out all previous activities, but able to look after own affairs without assistance”) (7, 8).

When possible, we stratified variables in continuous and categorical, analyzing each means e medians. The Kolmogorov–Smirnov test with Lilliefors significance correction was performed to test the normality of continuous variables. Statistical analyses were carried out using IBM SPSS Statistics for Mac OS, version 22.0 (IBM Corp., Armonk, NY, United States). Schematic figures were realized with BioRender^©^.

Written informed consent was obtained from all patients or their representatives. The study conformed to the Declaration of Helsinki of the World Medical Association and it was approved by the local ethics committee (Ref. No. CEUR-2020-Os-173).

## 3 Results

### 3.1 Demographic characteristics

We found 15 patients with IIS out of 563 ischemic stroke cases (15/563–2.6%). The mean age was 77 years (range, 35–89 years). There was not any prevalence regarding patients’ gender (8 males, 7 females) (Table 1).

**Table 1:** Demographic data and medical history of patients.

Patient ID	Demographic data		Medical history and comorbidities							
	Gender	Age (years)	Hypertension	Diabetes	Dyslipidaemia	Active smoke	BMI	Previous MI	Previous stroke	Leukoaraiosis
1	Female	75	Yes	No	No	No	48	No	No	No
2	Female	71	Yes	No	No	No	33	No	No	No
3	Male	80	No	No	No	No	26	No	No	Yes
4	Female	80	Yes	No	Yes	No	38	No	No	Yes
5	Male	67	No	No	Yes	No	31	No	No	Yes
6	Male	83	Yes	Yes	No	No	29	Yes	No	No
7	Male	35	No	No	Yes	No	34	No	No	No
8	Male	77	No	No	Yes	No	27	No	No	No
9	Male	87	Yes	No	Yes	No	28	No	No	No
10	Female	73	No	No	No	No	22	No	No	Yes
11	Female	88	Yes	No	No	No	21	No	Yes	No
12	Female	88	Yes	No	Yes	No	20	No	No	Yes
13	Male	61	No	No	No	No	26	No	No	No
14	Male	89	Yes	No	Yes	No	24	Yes	No	No
15	Female	63	No	Yes	No	No	23	No	No	No
Overall n (%) *or* median	Female, 7 (46.7%)	77 [35–89]	Yes, 8 (53.3%)	Yes, 2 (13.3%)	Yes, 7 (46.7%)	Yes, 0 (0%)	29 ± 7	Yes, 2 (13.3%)	Yes, 1 (6.6%)	Yes, 5 (33.3%)
[IQR] *or* mean ± sd	Male, 8 (53.3%)		No, 7 (46.7%)	No, 13 (86.7%)	No, 8 (53.3%)	No, 15 (100%)		No, 13 (86.7%)	No, 14 (93.4%)	No, 10 (66.7%)

BMI, body mass index; MI, myocardial infarction.

### 3.2 Medical history and comorbidities

The most frequent comorbidity was hypertension (8/15–53.3%), followed by dyslipidemia (7/15–46.7%). Two patients had diabetes mellitus type 2 (13.3%). Evidence of leukoaraiosis was found in five patients (33.3%): of them, only one patient has already been diagnosed with leukoaraiosis (1/15–6.6%), while in 26.6% (4/15) of cases it was discovered at the time of the AIS. We found one patient with history of previous ischemic stroke (6.6%) and two with previous acute myocardial infarction (13.3%). Most subjects presented body mass index (BMI) over the normal weight interval (mean ± sd, 29 ± 7). None of the patients was a smoker (Table 1).

### 3.3 Cardiological characteristics

**Figure 1:**
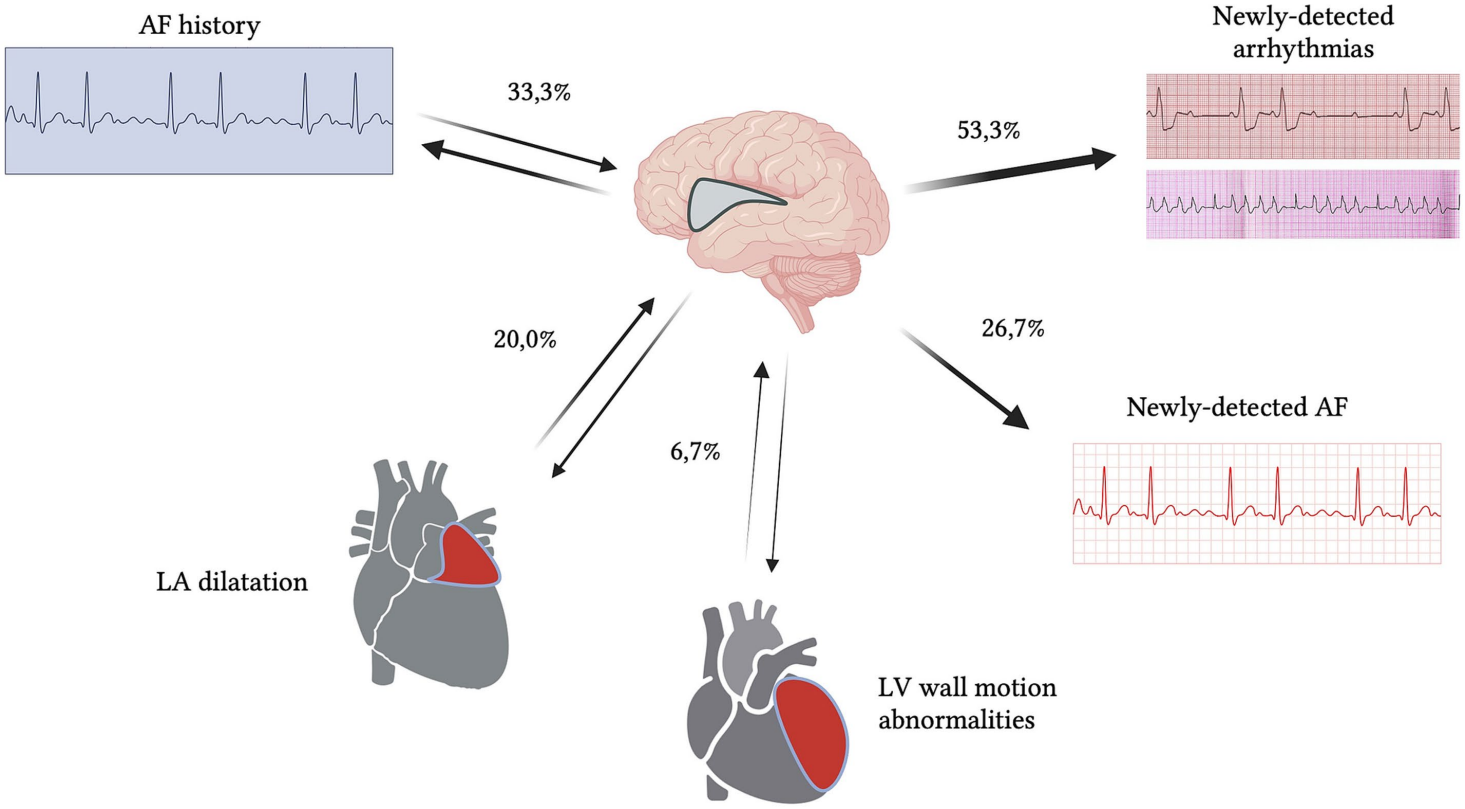
Schematic representation of cardiological characteristics concerning ECG and echocardiogram findings (percentages refer to Tables 2, 3). 26.7% of patients had newly detected atrial fibrillation (AF) and 53.3% of patients had other types of newly detected arrhythmias. Cardiovascular alterations were the most prevalent atypical etiology of isolated insular stroke (IIS) patients.

Five patients had history of atrial fibrillation (AF) (33.3%). We found four patients with newly detected AF during the hospitalization (26.6%). The other most reported arrhythmias were atrioventricular block (3/15–20.0%), ventricular extrasystoles (4/15–26.6%) and right bundle branch block (2/15–13.3%). Of the three patients with atrioventricular block (AVB), two had first grade AVB with mean PR interval of 295 ms, while another patient had type I second grade AVB (mean PR intervals ranged between 180 to 455 ms). We collected specific data from ECGs performed in the emergency room (ER) setting at admission: the mean value of QT corrected with Bazett formula (QTcB) was 443 ± 58 ms, while mean RR interval was 725 ± 255 ms. Five patients were already on treatment with antiarrhythmic oral therapy (33.3%). One patient needed intravenous antiarrhythmic therapy for persistent AF with fast ventricular rate. At admission, eight subjects (53.3%) were already on treatment with antihypertensive oral therapy. The median blood pressure detected in ER was 160/90 mmHg. We found three patients with hypertensive crisis, defined as systolic blood pressure (SBP) > 180 mmHg and/or diastolic blood pressure (DBP) > 120 mmHg (20.0%). Of these patients, one received intravenous antihypertensive therapy, due to persistent elevated SBP and DBP values. Echocardiography was performed in 9/15 patients (60.0%): left ventricular ejection fraction (LVEF) mean value was 61 (±10 standard deviation). Three patients presented mildly reduced LVEF (LVEF range 48–54%) (3/15–20.0%); one had wall motion abnormalities (6.6%) while other three had severe left atrial dilatation (20.0%) (Figure 1; Tables 2, 3).

**Table 2:** Cardiological characteristics concerning heart rhythm and blood pressure.

Patient ID	Cardiological characteristics										
	Rhythm							Blood pressure			
	AF history	Newly-detected AF	Other abnormal rhythm findings	Antiarrhythmic home therapy	R-R interval (ms)	QTcB (ms)	IV antiarrhythmic therapy	Antihypertensive home therapy	SBP/DBP (mmHg)	Hypertensive crisis during hospitalization	IV antihypertensive therapy
1	Yes	No	No	Yes	714	447	No	Yes	na	Yes	No
2	No	Yes	Mobitz type I second grade AVB	No	316	na	No	Yes	165/100	No	No
3	No	Yes	First grade AVB, VES	No	942	491	No	No	150/90	No	No
4	Yes	No	No	Yes	902	425	No	Yes	160/80	No	No
5	No	No	No	No	429	294	No	No	155/80	No	No
6	Yes	No	RBBB	Yes	333	485	No	Yes	160/75	No	No
7	No	No	VES	No	737	461	No	No	na	No	No
8	No	No	No	No	853	420	No	No	175/100	No	No
9	No	Yes	No	No	1,008	404	No	Yes	160/85	Yes	Yes
10	Yes	No	No	Yes	800	549	No	No	188/107	No	No
11	Yes	No	No	Yes	965	437	No	Yes	175/80	No	No
12	No	No	CAM	No	819	401	No	Yes	156/96	Yes	No
13	No	No	VES, SVES	No	908	451	No	No	145/90	No	No
14	No	No	First grade AVB, RBBB, LAFB	No	870	483	No	Yes	150/95	No	No
15	No	Yes	No	No	280	457	Yes	No	na	No	No
Overall n (%) *or* median	Yes, 5 (33.3%)	Yes, 4 (26.7%)	Yes, 8 (53.3%)	Yes, 5 (33.3%)	725 ± 255	443 ± 58	Yes, 1 (6.7%)	Yes, 8 (53.3%)	SBP 160 [145–188], DBP 90 [75–107]	Yes, 3 (20.0%)	Yes, 1 (6.7%)
[IQR] *or* mean ± sd	No, 10 (66.7%)	No, 11 (73.7%)	No, 7 (46.7%)	No, 10 (66.7%)			No, 14 (93.3%)	No, 7 (46.7%)		No, 12 (80.0%)	No, 14 (93.3%)

AF, atrial fibrillation; AVB, atrioventricular block; VES, ventricular extrasystoles; SVES, supraventricular extrasystoles; RBBB, right bundle branch block; CAM, chaotic atrial rhythm; LAFB, left anterior fascicular block; QTcB, QT corrected with Bazett formula; SBP, systolic blood pressure; DBP, diastolic blood pressure; na, not available.

**Table 3:** Cardiological characteristics concerning echocardiographic findings.

Patient ID	Cardiological characteristics		
	Echocardiographic findings		
	LVEF (%)	LV wall motion abnormalities	LA dilatation
1	–	–	–
2	61	No	Severe
3	–	–	–
4	–	–	–
5	72	No	No
6	48	Yes (medio-basal inferior wall hypokinesia-akinesia)	No
7	–	–	–
8	54	No	No
9	56	No	Severe
10	51	No	No
11	–	–	–
12	77	No	Severe
13	69	No	No
14	59	No	No
15	na	na	na
Overall *n* (%) *or* median	61 ± 10	Yes, 1 (6.7%)	Yes, 3 (20.0%)
		No, 8 (53.3%)	No, 6 (40.0%)
[IQR] *or* mean ± sd		na, 1 (6.7%)	na, 1 (6.7%)

LVEF, left ventricular ejection fraction; LV, left ventricle; LA, left atrium; na, not available.

### 3.4 Clinical symptoms

**Figure 2:**
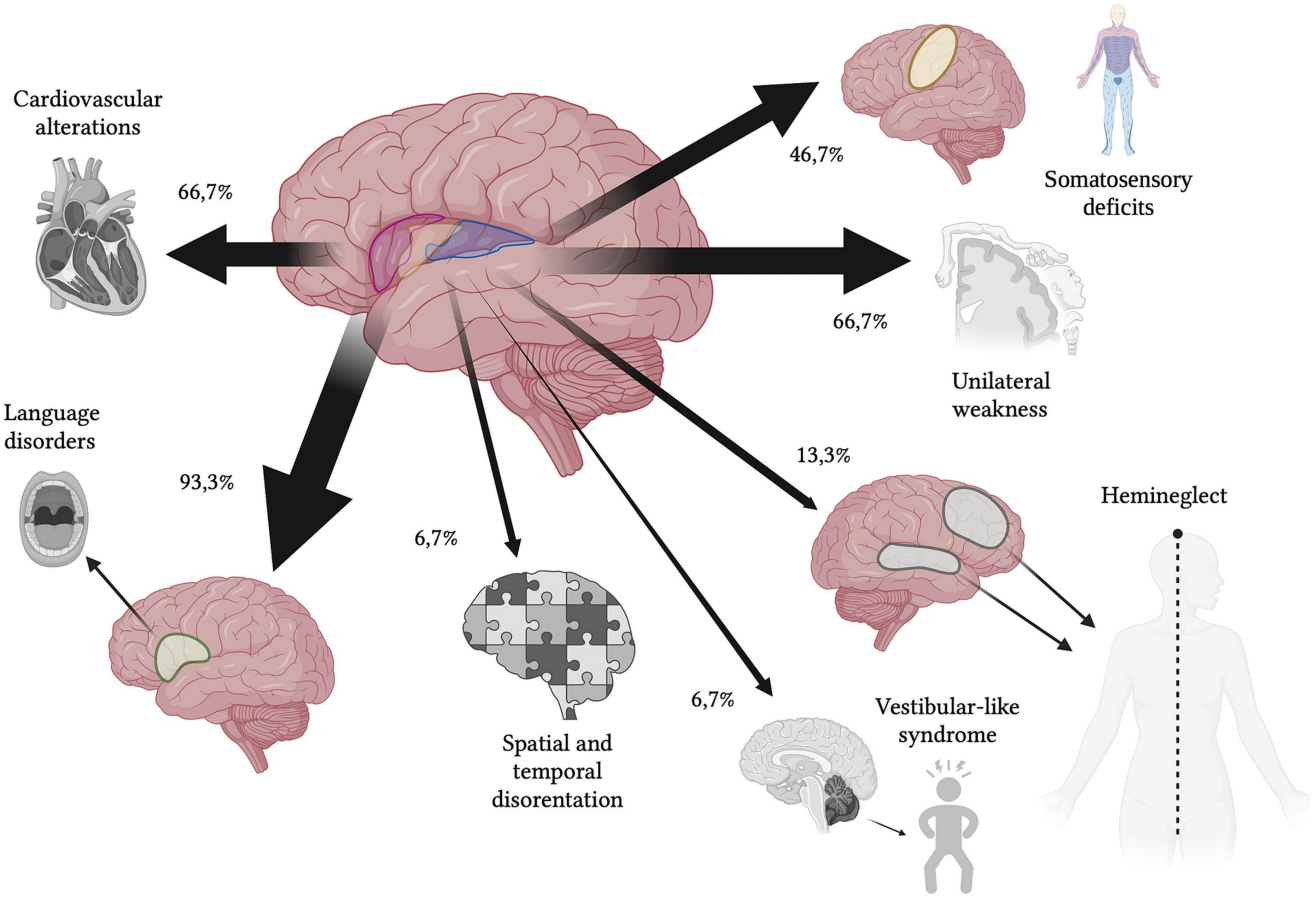
Schematic representation of clinical symptoms (percentages refer to Table 4). In order of frequency, typical symptoms were language disorder (93.3%), motor deficits (66.7%), somatosensory deficits (46.7%) and vestibular-like syndrome (6.7%), while atypical symptoms were newly detected cardiovascular alterations (66.7%), spatial and awareness deficits (13.3%), space and time disorientation (6.7%) and headache (6.7%).

All the 15 patients presented typical symptoms (100%). The most frequent manifestation was language disorder (14/15–93.3%), followed by motor deficits (10/15–66.6%) and somatosensory deficits (7/15–46.6%). Eleven patients presented atypical symptoms (11/15–73.7%), particularly the majority with newly-detected cardiovascular alterations (10/15–66.6%), followed by spatial and awareness deficits (2/15–13.3%), space and time disorientation (1/15–6.7%) and headache (1/15–6.7%). Only one patient presented vestibular-like syndrome (1/15–6.7%) categorized in the typical symptoms group (Figure 2; Table 4).

**Table 4:** Topography of lesion, clinical symptoms, stroke etiology, and reperfusion treatment.

Patient ID	Topography of lesion		Clinical symptoms		Stroke etiology	Reperfusion treatment
	Site and side	LVO	Typical symptoms	Atypical symptoms	TOAST classification	Type of procedure
1	TIC, right hemisphere	M1	Unilateral weakness, somatosensory deficits	Hemineglect, cardiovascular alterations	CE	MT
2	TIC, left hemisphere	No	Somatosensory deficits, non-fluent aphasia	Cardiovascular alterations	CE	IVT
3	AIC, left hemisphere	M2	Unilateral weakness, global aphasia	Cardiovascular alterations	CE	IVT + MT
4	PIC, left hemisphere	M2-M3	Fluent aphasia	Temporal and spatial disorientation	CE	None
5	PIC, left hemisphere	M2	Global aphasia	-	UND	IVT + MT
6	PIC, left hemisphere	M2	Unilateral weakness, somatosensory deficits, global aphasia	Cardiovascular alterations	LAD	MT
7	AIC, left hemisphere	M2	Unilateral weakness, non-fluent aphasia	Cardiovascular alterations	UND	IVT + MT
8	TIC, right hemisphere	No	Unilateral weakness, somatosensory deficits, dysarthria	–	UND	IVT
9	AIC, left hemisphere	M1	Unilateral weakness, global aphasia	Cardiovascular alterations	CE	IVT + MT
10	AIC, left hemisphere	M1-M2	Unilateral weakness, non-fluent aphasia	–	CE	MT
11	TIC, left hemisphere	M1	Unilateral weakness, somatosensory deficits, global aphasia	–	LAD	MT
12	TIC, left hemisphere	No	Global aphasia	Cardiovascular alterations	UND	None
13	PIC, left hemisphere	No	Transient somatosensory deficits, dysarthria, vestibular-like syndrome	Headache, cardiovascular alterations	CE	None
14	PIC, left hemisphere	M1	Unilateral weakness, dysarthria	Cardiovascular alterations	UND	IVT + MT
15	TIC, right hemisphere	M1-M2	Unilateral weakness, somatosensory deficits, dysarthria	Hemineglect, cardiovascular alterations	CE	IVT + MT
Overall n (%) *or* median	AIC, 4 (26.6%)	Yes, 11 (73.3%)	Unilateral weakness, 10 (66.7%)	Hemineglect, 2 (13.3%)	LAD, 2 (13.3%)	IVT, 2 (13.3%)
[IQR] *or* mean ± sd	PIC, 5 (33.3%)		Somatosensory deficits, 7 (46.7%)	Temporal and spatial disorientation, 1 (6.7%)	CE, 8 (53.3%)	MT, 4 (26.6%)
	TIC, 6 (40.0%)	No, 4 (26.7%)	Aphasia, 10 (66.7%)	Cardiovascular alterations, 10 (66.7%)	UND, 5 (33.3%)	IVT + MT, 6 (40.0%)
	Left, 12 (80.0%)		Dysarthria, 4 (26.7%)	Headache, 1 (6.7%)		None, 3 (20.0%)
	Right, 3 (20.0%)		Vestibular-like syndrome, 1 (6.7%)			

AIC, anterior insular cortex; PIC, posterior insular cortex; TIC, total insular cortex; LVO, large vessel occlusion; M1, horizontal segment of middle cerebral artery; M2, sylvian segment of middle cerebral artery; M3, cortical segment of middle cerebral artery; CE, cardioembolic; LAD, large artery disease; UND, undetermined; IVT, intravenous thrombolysis; MT, mechanical thrombectomy.

### 3.5 Site and side of lesion

**Figure 3:**
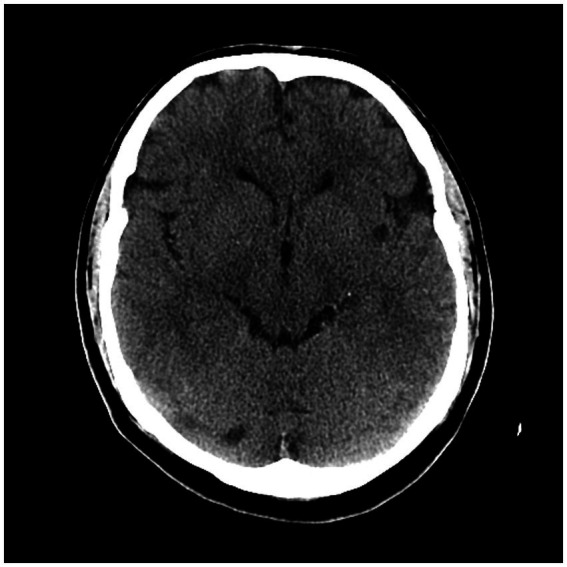
CT scan showing left posterior insular cortex (PIC) ischemic lesion.

**Figure 4:**
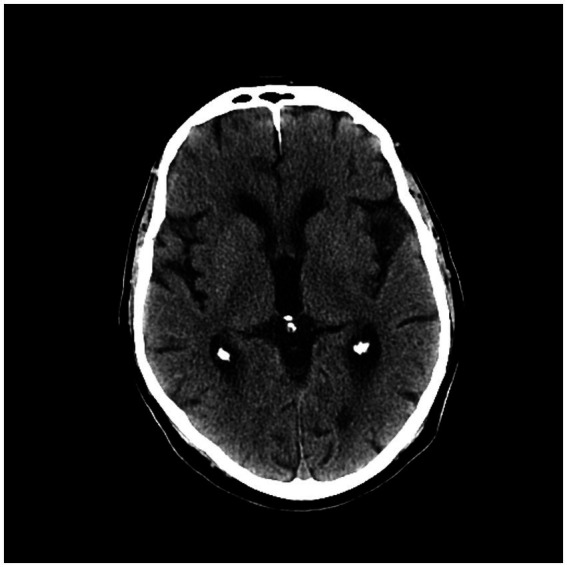
Second CT scan showing left posterior cortex (PIC) ischemic lesion.

**Figure 5:**
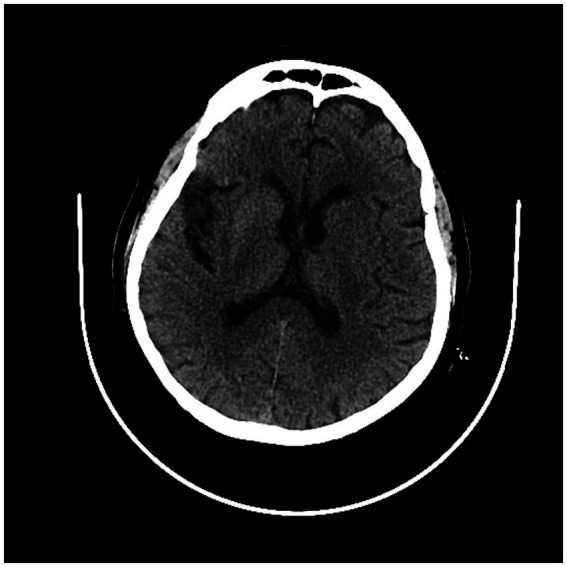
CT scan showing right total insular cortex (TIC) ischemic lesion.

We found 12 patients with left IIS (80%) and only three patients with right IIS (20.0%). We detected 4/15 (26.6%) patients with anterior insular cortex lesion (AIC), 5/15 (33.3%) with posterior insular cortex lesion (PIC) and 6/15 (40%) with total insular cortex lesion (TIC). Eleven subjects (73.7%) had middle cerebral artery (MCA) involvement at CT angiography (CTA) (73.3%): 6/15 patients (%) had prevalent M1 segment occlusion, and 5/15 (33.3%) had prevalent M2 segment occlusion. The other four patients (26.6%) had no visible large vessel occlusion at CTA (Figures 3–5; Table 4).

### 3.6 Etiology

According to the TOAST classification (9), cardiac embolism was the most frequent etiology (8/15–53.3%); 2/15 patients had large artery disease (LAD) (13.3%), while five had a cryptogenic stroke (30.0%) (Table 4).

### 3.7 Reperfusion treatment

Two patients received only intravenous thrombolysis (2/15–13.3%) and four received only mechanical thrombectomy (4/15–27%). Six subjects received bridging therapy (rT-PA plus MT) (6/15–40%). The remaining patients did not meet the criteria for thrombolysis protocol, based on International Guidelines for reperfusion therapy (Table 4).

### 3.8 Outcome measures

The median NIHSS before reperfusion therapy was 11 (range 0–22) and the median NIHSS at discharge was 0 (range 0–22). Of note, five patients had persistent aphasic disorder at discharge (33.3%). The median delta NIHSS (defined as the difference between NIHSS before therapy and NIHSS at discharge) was 6. Most patients had major neurological improvement at discharge (defined as improvement of 8 points on the NIHSS from baseline or a NIHSS score of 0 or 1 at discharge) (13/15–86.7%).

The majority of patients did not have disability before the event, with median mRS score 0 (range 0–4). At discharge, median mRS score was 2 (range 0–4), while median 3-months mRS score was 0 (range 0–4). Good outcome (defined as 3-months mRS score of 0–2; for patients with pre-stroke mRS score > 2, return to the pre-stroke mRS three months after acute ischemic stroke-AIS-) was observed in most cases (mRS 0–2: 14/15–93.3%) (Table 5).

**Table 5:** Outcome measures.

Patient ID	Outcome measures							
	Incoming NIHSS	NIHSS at discharge	Major neurological improvement	Language disorder at discharge	Pre-stroke mRS	mRS at discharge	3-months mRS	Good outcome
1	16	3	Yes	No	2	4	3	No
2	2	0	Yes	No	0	0	0	Yes
3	18	0	Yes	No	0	2	0	Yes
4	2	1	Yes	Yes	3	4	3	Yes
5	9	5	No	Yes	0	2	1	Yes
6	22	0	Yes	No	2	2	2	Yes
7	8	0	Yes	No	0	0	0	Yes
8	4	0	Yes	No	0	0	0	Yes
9	11	5	No	Yes	0	0	0	Yes
10	17	3	Yes	Yes	0	4	0	Yes
11	22	22	No	Yes	4	4	4	Yes
12	6	0	Yes	No	0	3	1	Yes
13	0	0	Yes	No	0	0	0	Yes
14	12	0	Yes	No	0	0	0	Yes
15	12	3	Yes	No	0	1	1	Yes
Overall n (%) *or* median	11 [0–22]	0 [0–22]	Yes, 13 (86.7%)	Yes, 5 (33.3%)	0 [0–4]	2 [0–4]	0 [0–4]	Yes, 14 (93.3%)
[IQR] *or* mean ± sd			No, 2 (13.3%)	No, 10 (66.7%)				No, 1 (6.7%)

86.7% of patients had major neurological improvement and 93.3% of patients had good outcome.

Major neurological improvement (improvement of 8 points on the NIHSS from baseline or a NIHSS score of 0 or 1 at discharge), good outcome (3-months mRS score of 0–2; for patients with pre-stroke mRS score > 2, return to the pre-stroke mRS 3 months after AIS).

## 4 Discussion

To our knowledge, the presented study is the largest monocentric case series of pure insular ischemic stroke. In fact, previously published case series by Lemieux et al. brilliantly presented a group of 23 patients of which 7 were recruited from their database and the remaining 16 were included from previously published cases, while Giammello et al. recently published an interesting single center-case series of 13 IIS focusing on their clinical presentation (9, 10).

Despite the increasing number of functional neuroimaging studies, the insular cortex wide array of functions remains a matter of interest and debate. In 2010, using the activation likelihood estimation (ALE) method, *Kurth and al.* tried to merge 13 insular cortex functional categories (i.e., emotion, empathy, olfaction, gustation, interoception, pain, somatosensation, motor, attention, language, speech, working memory and memory) into four functional domains, based on the connections with the adjacent brain structures: (1) a sensori-motor domain in the mid-posterior insula; (2) a social–emotional domain in the anterior-ventral insula; (3) a cognitive domain in the anterior-dorsal insula; (4) an olfactory-gustatory domain in the middle insular gyrus (2). It goes without saying that an isolated insular lesion, such as an ischemic stroke, could manifest with several different clinical presentations. Di Stefano et al. suggested to categorize symptoms in “typical” and “atypical,” according to the frequency of IIS case reports and case series clinical presentations (i.e., “typical” symptoms for motor and somatosensory deficits, dysarthria, aphasia, and vestibular-like syndrome; “atypical” symptoms for dysphagia, awareness deficits, gustatory disturbances, dysautonomia, neuropsychiatric or auditory disturbances and headache) (5). A combination of these symptoms-especially the typical ones-is common in the same patient. In our study, all the patients presented with typical symptoms, alone or in combination, with a predominance of language disorders (aphasia and dysarthria), while 11/15 patients presented with atypical symptoms, mainly represented by abnormal cardiac findings (10/11 patients). Precisely, AF was newly detected during the hospitalization in 4 patients, as well as ventricular extrasystoles (3/15), atrioventricular blocks (3/15), right bundle branch blocks (2/15), supraventricular extrasystoles (1/15), chaotic atrial rhythm (1/15), and left anterior fascicular block. Of note, none of these patients were under antiarrhythmic home therapy at the time of stroke onset. It is known the key role of the insula in regulating autonomic functions, such as heart rate, cardiac rhythm and blood pressure (11). Thus, it has been hypothesized that an ischemic insular lesion could imbalance the autonomic cardiac control, resulting in reduced parasympathetic tone and increased sympathetic tone, leading to catecholamine-related myocardial toxicity and manifesting with tachyarrhythmias, ventricular dysfunction, and myocardial infarction (11–13). Particularly, different studies identified an association between insular stroke and new-onset AF, supporting this neurogenic hypothesis, especially in the absence of a previous cardiac disease or abnormal cardiac imaging (14, 15). In our cohort, two out of four patients diagnosed with new-onset AF had a severe left atrial dilatation on echocardiogram, suggesting therefore a possible cardiogenic nature of AF rather than a neurogenic one. Nonetheless, clearly distinguishing between the two etiologies in the clinical setting remains challenging (14). As patients’ cardiac rhythm monitoring lasted meanly 48 h, we cannot exclude that a longer cardiac monitoring might have detected a higher percentage of arrythmias. Literature regarding long cardiac monitoring in insular stroke patients is unfortunately scarce, however a recent position paper written by AF-SCREEN International Collaboration proposed continuous ECG monitoring for at least 72 h after the index event in patients with TIA or ischemic stroke to detect AF. Concerning the other ECG abnormalities we found in our patients, *Christensen* et al. demonstrated that insular lesions are related to changes affecting RR interval, PR interval, ST segment, QTc and T-wave (16). Surprisingly, the mean values of RR interval and QTcB at the time of admission resulted within the normal ranges, although a heart rate variability analysis was not performed. Regarding IIS etiology, according to *Stefano* et al. review, embolic occlusion of M2 or its branches is the main cause of IIS (even though the origin of embolism is prevalently cryptogenic), while the most prevalent known etiology appears to be large-artery disease, followed by cardioembolism (CE) (5). In our case series, the most prevalent etiology resulted in CE (8/15). Indeed, a retrospective large study conducted by a Korean group in 2015 found that the frequency of CE was significantly higher than that in patients without insular involvement (17). Although the Korean study considered patients with ischemic lesions involving the insular cortex and not exclusively restricted to it, a full diagnostic work-up for the research of CE sources should be considered. In fact, if hyper-acute management of insular stroke does not differ from the management of other stroke localizations, nonetheless, after the acute phase, cardiac activity should be closely monitored. Conversely, as in these patients AF may not necessarily be the cause of stroke, but a consequence of acute brain injury, searching for laboratory (pro-BNP) and echocardiographic (left atrial dilation) signs of atrial cardiomyopathy may be useful to distinguish between the two conditions. Finally, it should be considered that in these patients new-onset AF could be a stroke consequence that resolves itself to stabilization of ischemia: thus, it may not deserve chronic anticoagulation.

Given the cardinal role of the insular cortex as a cerebral integration hub, the IIS outcome should be expected to be extremely unfavorable: conversely, most case report and case series patients had a general good recovery and low mRS (5, 9, 10). Our data confirm this trend as more than 80% of the patients had major neurological improvement at discharge (defined as improvement of 8 points on the NIHSS from baseline or a NIHSS score of 0 or 1 at discharge) and more than 90% of the patients had good outcome, defined as 3-months mRS score of 0–2 (for patients with pre-stroke mRS score > 2, return to the pre-stroke mRS three months after AIS), with median 3-months mRS score of 0 (range 0–4). Regarding NIHSS at discharge, it is interesting to point out that the highest scores might be linked to the persistence of an isolated aphasic disorder at discharge. Although there is limited evidence, due to the absence of long-term data on IIS follow-up and to its extreme low frequency, there are several possible reasons which could explain the general good outcome of IIS. First, as suggested by Lemieux et al. (9), the insula is by definition a restricted anatomic region of which the surrounding structures could compensate a limited damage, as demonstrated by tumor resection and epilepsy surgery studies (18, 19); second, as the insular cortex is predominantly vascularized by M2 segment of MCA and its divisions, with a small contribution by the insular branches of M1 segment and without the supply of pial collateral anterior and posterior circulation (20, 21), an IIS is an extremely rare occurrence while an insular cortex involvement in a MCA occlusion is very common and it is considered to be a marker of negative outcome and large infarct stroke (3, 22, 23). Thus, it has been hypothesized that most of the clinical deficits are due to an hypoperfusion of the MCA territory, which might be reversible after MCA recanalization (3, 24, 25). In our case series, of 11 patients with evidence of LVO (M1 or M2), 10 had received MT and 10/11 showed 3-months good outcome. In addition, the possible presence of anatomical variants leading to insular poor vascularization has not been yet considered or deepened in previous studies, nor the presence of anatomical variants have emerged in our patients’ CT angiographies.

The present study has several limitations. First, the retrospective nature of the study could have biased the data collection and the results; second, a complete heart rate variability analysis could have added information on cardiological aspects linked to a possible shift in balance toward an augmented sympathetic tone (11); third, there are no follow-up data concerning ECGs and echocardiograms of our patients, which could have supported the neurogenic hypothesis in the genesis of new-onset arrythmias.

## 5 Conclusion

IIS are extremely rare, representing according to our study about 2.6% ischemic strokes cases per year, and patients have peculiar clinical manifestations, such as dysautonomia and awareness deficits. Our data suggest the possibility for these patients to completely recover after AIS, as more than 80% of our patients had major neurological improvement at discharge and more than 90% had low mRS at three months after the event, notwithstanding the pivotal role of the insula in cerebral connections and the frequent association with MCA occlusion. Moreover, given the central role of the insula in regulating autonomic functions, cardiac alterations must be taken into consideration, as well as a full diagnostic work-up for the research of cardioembolic sources should be mandatory. Prospective studies are needed to better define these aspects. To our knowledge, the presented study is the largest monocentric case series of pure insular stroke and it might be useful for future systematic reviews.

## Data availability statement

The original contributions presented in the study are included in the article/supplementary material, further inquiries can be directed to the corresponding authors.

## Ethics statement

The studies involving humans were approved by Comitato Etico Unico Regionale Fvg (CEUR) Palazzina C, Piano Terra, Via Pozzuolo 330, Udine Ref. No. CEUR-2020-Os-173. The studies were conducted in accordance with the local legislation and institutional requirements. The participants provided their written informed consent to participate in this study. Written informed consent was obtained from the individual(s) for the publication of any potentially identifiable images or data included in this article.

## Author contributions

FK: Data curation, Formal analysis, Investigation, Methodology, Project administration, Visualization, Writing – original draft, Writing – review & editing. ST: Data curation, Formal analysis, Software, Writing – review & editing. RS: Data curation, Writing – review & editing. LC: Data curation, Writing – review & editing. DB: Investigation, Writing – review & editing. SL: Conceptualization, Writing – review & editing. GM: Validation, Writing – review & editing. FJ: Writing – review & editing. CG: Writing – review & editing. RM: Writing – review & editing. LV: Writing – review & editing. MV: Supervision, Validation, Writing – review & editing. GP: Conceptualization, Supervision, Validation, Visualization, Writing – original draft, Writing – review & editing.
